# Diffany: an ontology-driven framework to infer, visualise and analyse differential molecular networks

**DOI:** 10.1186/s12859-015-0863-y

**Published:** 2016-01-05

**Authors:** Sofie Van Landeghem, Thomas Van Parys, Marieke Dubois, Dirk Inzé, Yves Van de Peer

**Affiliations:** Department of Plant Systems Biology, VIB, Technologiepark 927, Ghent, 9052 Belgium; Department of Plant Biotechnology and Bioinformatics, Ghent University, Technologiepark 927, Ghent, 9052 Belgium; Genomics Research Institute, University of Pretoria, Private bag X200028, Pretoria, South Africa

**Keywords:** Differential networks, Osmotic stress response, Systems biology

## Abstract

**Background:**

Differential networks have recently been introduced as a powerful way to study the dynamic rewiring capabilities of an interactome in response to changing environmental conditions or stimuli. Currently, such differential networks are generated and visualised using ad hoc methods, and are often limited to the analysis of only one condition-specific response or one interaction type at a time.

**Results:**

In this work, we present a generic, ontology-driven framework to infer, visualise and analyse an arbitrary set of condition-specific responses against one reference network. To this end, we have implemented novel ontology-based algorithms that can process highly heterogeneous networks, accounting for both physical interactions and regulatory associations, symmetric and directed edges, edge weights and negation. We propose this integrative framework as a standardised methodology that allows a unified view on differential networks and promotes comparability between differential network studies. As an illustrative application, we demonstrate its usefulness on a plant abiotic stress study and we experimentally confirmed a predicted regulator.

**Availability:**

Diffany is freely available as open-source java library and Cytoscape plugin from http://bioinformatics.psb.ugent.be/supplementary_data/solan/diffany/.

**Electronic supplementary material:**

The online version of this article (doi:10.1186/s12859-015-0863-y) contains supplementary material, which is available to authorized users.

## Background

In the early days of Systems Biology, when molecular interaction data was still relatively sparse, all interactions known for a model organism were typically added to a single large interaction network. Such an integrated view would combine data from the proteome, transcriptome and metabolome [1–4]. While such studies certainly proved valuable to gain insights into the general characteristics of molecular networks, they lack the level of detail required to analyse specific response mechanisms of the interactome to changing conditions or stimuli. Consequently, differential networks have been introduced to model the dynamic rewiring of the interactome under specific conditions [5, 6]. Differential networks only depict the set of interactions that changed after the introduction of a stimulus. Most current research in this field has focused on a single interaction type such as expression data [7, 8], genetic interactions [9] or protein complexes [10]. Further, the analysis is usually limited to the comparison of only two networks [11–13]. At the same time, several promising studies have constructed multiple condition-specific networks such as time-course data [14, 15], tissue-specific networks [16, 17] or stress-induced co-expression networks [18]. These studies analyse general network statistics such as connectivity scores or employ machine-learning techniques to identify significantly rewired genes. However, due to the black-box behaviour of the methods and because these studies do not actually generate and visualise differential networks, the resulting prioritised gene lists cannot be easily interpreted by domain experts. By contrast, we believe it to be crucial that researchers can visualise and further explore the rewiring events in their network context. Unfortunately, there is currently no standardised methodology that would allow to integrate heterogeneous condition-specific networks on the one hand, and produce intercomparable differential networks on the other hand.

Here, we introduce a novel ontology-based framework to standardise condition-specific input networks and to allow an arbitrary number of such networks to be used in the inference of a differential network. The network algorithms are designed to cope with a high variety of heterogeneous input data, including physical interactions and regulatory associations, symmetric and directed edges, explicitly negated interactions and edge weights. Depending on the application, these weights may be used to model the strength of an interaction, determined for instance by the expression levels of the interacting genes, or they may represent the probability that an interaction occurs when dealing with computationally inferred networks such as regulatory associations derived from co-expression analysis.

To the best of our knowledge, our integrative framework named ‘Diffany’ (Differential network analysis tool) is unique in the emerging field of differential network biology, and we hope its open-source release will facilitate and enhance differential network studies. As one such example, we will present how the reanalysis, with Diffany, of a previously published experimental dataset has unveiled a novel candidate regulator for plant responses to mannitol. Experimental validation confirmed that this regulator, HY5, might indeed be involved in the mannitol-responsive network in growing Arabidopsis leaves.

## Framework

In this section, we detail the various parts of the Diffany framework (Additional file 1).

### Network terminology

To perform a differential network analysis, two types of input data sources are required. First, a *reference networkR* models an untreated/unperturbed interactome, serving as the point of reference to compare other networks to. Second, one or more *condition-specific networks* each represent the interactome after a certain treatment, perturbation or stimulus. We denote them as *N*_*i*_ with *i* between 1 and *c*, and *c* the number of distinct conditions that are being compared to the reference state.

Both types of input networks may have edges with a certain *weight* associated to them. Such weights in the networks may be interpreted differently according to the application for which the framework is used. For instance, they may model the strength of physical interactions as determined by expression levels of the interacting genes. In other cases, when dealing with network data inferred through computational methods, such as regulatory associations derived from co-expression data, these weights may instead model the probability/confidence that an interaction really does occur. Whichever the case, the Diffany framework assumes the weights assigned to the edges are sensible and comparable to each other.

**Fig. 1:**
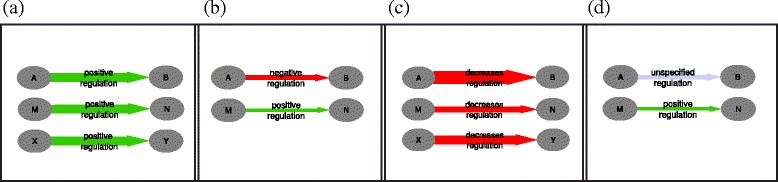
Differential edges. Artificial example of the inference of differential edges (**c**) from a reference network (**a**) and a condition-specific network (**b**). Edge thickness refers to the weight of an edge. In Subfigure (**c**), the top connection (A-B) shows a negative differential edge (‘decreases_regulation’) occurring because of a switched polarity from positive (green) to negative (red) regulation, while the second and third links (M-N and X-Y) show a negative differential edge because the original positive edge was decreased or even entirely removed in the condition-specific network. The thickness of the differential edge represents the difference in weight between the reference and condition edge. Column (**d**) depicts the corresponding ‘consensus’ edges: both input networks are found to have a regulatory edge between nodes A and B and a positive regulation edge between M and N, but there is no consensus edge between X and Y

The two input sources are used to generate a *differential networkD* (Fig. 1) that depicts the rewiring events from the reference state to the perturbed interactome. Further, an inferred *consensus networkC* models the interactions that are common to the reference and condition-specific networks, sometimes also called ‘housekeeping’ interactions. We do not adopt the latter terminology, because while some unchanged interactions may indeed provide information about the cell’s standard machinery (i.e. housekeeping functions), others may simply refer to interactions that change under some other condition than the one tested in the experimental setup.

### Interaction ontology

The interaction ontology is a crucial component that assigns meaning to heterogeneous input data types. Analogous to the Systems Biology Graphical Notation (SBGN) [19], this structured vocabulory provides a distinction between ‘Activity Flow’ interactions and ‘Process’ interactions, modelling regulatory associations and physical interactions separately. However, in contrast to SBGN, these complementary interaction classes can be freely mixed within one network, allowing for a varying level of modelling detail combined into one visualisation.

**Fig. 2:**
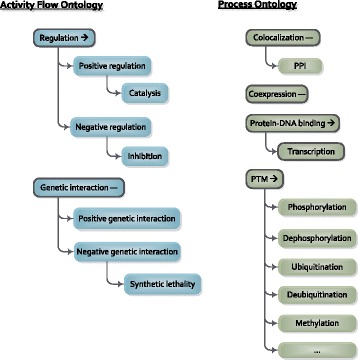
Interaction ontology. Default edge ontology structure, with activity flow interaction types on the left, and process types on the right. Root categories are shown with black borders, and have a default symmetry state: directed (→) or symmetrical (-). Because of space constraints, not all PTM (post-translational modification) subclasses are shown

In the Diffany framework, a default interaction ontology is available, covering genetic interactions, regulatory associations, co-expression, protein-protein interactions, and post-translational modifications (Fig. 2). This ontology was composed specifically to support a wide range of use-cases, and is used throughout this paper. However, the ontology structure itself, as well as the mapping of spelling variants, can be extended or modified based on specific user demands. Additionally, when unknown interaction types are encountered in the input data, they are transparently added as unconnected root categories.

### Network inference

The interaction ontology defines the root categories for which consensus and differential edges can be inferred. For the sake of simplification of the formulae in the following, we define *R*=*N*_0_, and we thus have a set $\mathcal {N}$ of *c*+1 input networks. The union of all nodes in these *c*+1 input networks is represented by $\mathcal {G}$, and an edge of semantic root category *S* between two nodes *X* and *Y* in an input network *N*_*i*_ as *I*_*sxyi*_. Notice that *I*_*sxyi*_ may also refer to a non-existing or ‘void’ edge when the two nodes *X* and *Y* are not connected by any edge of that semantic category *S* in the network *N*_*i*_.

A differential network is then inferred by considering each possible node pair (*X*,*Y*) in ($\mathcal {G} \times \mathcal {G}$) and, for each such pair, constructing the set of input edges $\mathcal {I}_{\textit {sxy}}$ for each semantic category *S*. The calculation of differential and consensus edges *E* from that set of input edges $\mathcal {I}_{\textit {sxy}}$ involves the determination of the following edge parameters:

- edge negation: *neg*(*E*) is a boolean value
- edge symmetry: *symm*(*E*) is a boolean value
- edge weight: *weight*(*E*) is a positive real number
- edge type: *type*(*E*) is a String value

#### Differential networks

**Fig. 3:**
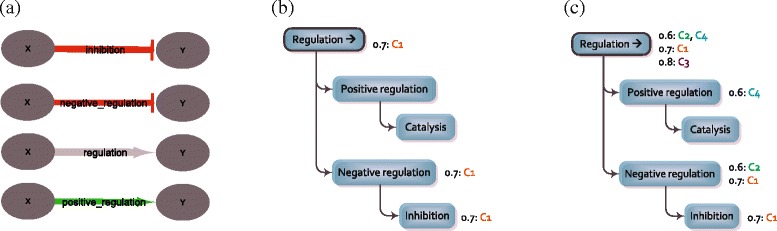
Evidence summarisation. Example of how the evidence from four different condition-specific networks ((**a**): C1-C4 from top to bottom) is summarised using the default edge ontology as backbone (shown only partially). Each condition-specific edge provides support not only for the category it belongs to (e.g. inhibition), but also for all super-categories in the tree (e.g. regulation (**b**)). After processing all condition-specific edges (**c**), the support tree summarises the number of condition-specific networks that support a certain category, and with which weights they do so

The hierarchical structure of the interaction ontology forms the backbone for the inference of differential networks. First, all (affirmative) condition-specific edges in $\mathcal {I}_{\textit {sxy}}$ for a specific category *S* are processed to construct a support tree (Fig. 3). Such an edge provides support not only for the category it belongs to (e.g. ‘inhibition’), but also for all super-categories in the tree (in casu, ‘negative regulation’ and ‘regulation’, cf. left tree in Fig. 3). From the support tree that is thus generated, it becomes possible to synthesize the number of condition-specific networks that support a certain category, and by which weights they do so (cf. right tree in Fig. 3).

Negated edges in $\mathcal {I}_{\textit {sxy}}$ are interpreted as explicit recordings of links that are not present in the interactome, but otherwise do not influence the support tree. A differential edge *D*_*sxy*_ is always affirmative (Formula (1)), and is only symmetrical when all input edges in $\mathcal {I}_{\textit {sxy}}$ are symmetrical (Formula (2)). When only some of the edges in $\mathcal {I}_{\textit {sxy}}$ are symmetrical while others are directed, the symmetrical ones are unmerged into two opposite directed edges of equal type and weight.

To further determine the type and weight of a differential edge *D*_*sxy*_, the reference edge *R*_*sxy*_ is compared to the produced support tree of the condition-specific networks. If the set of values in the support tree (e.g. {0.6, 0.7, 0.8} for ‘regulation’) contains values both below as well as above the weight of *R*_*sxy*_, no meaningful differential edge *D*_*sxy*_ can be deduced, as the response varies in directionality between the different conditions. This is also the case when the edges in $\mathcal {C}_{\textit {sxy}}$ all appear to be equal to *R*_*sxy*_. Otherwise, when all conditions support a higher weight than the weight of *R*_*sxy*_, the minimal difference to those supporting edges determines the increase value shared among all conditions and is thus used as the weight of *D*_*sxy*_ (Formula (3)). Similarly, when all conditions support a lower weight, the minimal difference determines the decrease value shared among all conditions. For example, if *R*_*sxy*_ would be a regulation edge of weight 0.9, *D*_*sxy*_ would be of type decrease_regulation and weight 0.1 according to the support tree of of Fig. 3. If *R*_*sxy*_ would have weight 0.4 instead, *D*_*sxy*_ would be of type increase_regulation and weight 0.2.

While a Process edge expresses a physical interaction and has no polarity, an Activity flow edge can be determined to have a general ‘positive’ or ‘negative’ effect. This means that for an edge in the Activity flow category (e.g. ‘positive regulation’) also edges of the opposite category can be compared (in casu ‘negative regulation’). While in principle edge weights are positive, in this case the weights of the opposite category will be converted to negative values only for calculation purposes. As such, the differential edge between ‘negative regulation’ of 0.2 (interpreted as −0.2 for calculation purposes) and ‘positive regulation’ of 0.3 would be of weight 0.5.

(1) $$ {neg(D_{sxy}) = false}  $$

(2) $$ {symm(D_{sxy}) = \bigwedge\limits_{i=0}^{c} symm(I_{sxyi})}  $$

(3) $$ {weight(D_{sxy}) = \min\limits_{i=1}^{c} \left(\left|weight(I_{sxy0}) - weight(I_{sxyi})\right|\right)}  $$

#### Consensus networks

The inference of consensus networks follows a similar procedure. To calculate a consensus edge *C*_*sxy*_ from a set of affirmative input edges $\mathcal {I}_{\textit {sxy}}$, the reference edge *R*_*sxy*_ is first added to the support tree in a similar fashion as done previously for the condition-specific edges. The most-specific edge type with highest weight that is supported by all input networks is then chosen to define the consensus edge. In the case when all edges in $\mathcal {I}_{\textit {sxy}}$ are negated, we construct a similar support tree, but one where the support travels downwards to sub-categories instead of upwards (e.g. ‘no regulation’ also implies ‘no inhibition’). In this case, the least-specific edge type with the highest weight that is supported by all, will represent the consensus edge, which will also be negated (Formula (4)). When $\mathcal {I}_{\textit {sxy}}$ contains both affirmative and negated edges, no consensus edge will be deduced between nodes *X* and *Y*.

As described above, consensus edges are defined by retrieving a weight value that is supported by all input, thus effectively applying a ‘minimum’ operator to the input weights (Formula (6)). However, it is also possible to apply the maximum operator, which will identify the highest weight that is supported by at least one input network, thus simulating a ‘union’ operation rather than an ‘intersection’ between the given input edges. More sophisticated weighting mechanisms will be implemented in the future, depending on the applications in which the framework will be used.

(4) $$ {neg(C_{sxy}) = \bigwedge\limits_{i=0}^{c} neg(I_{sxyi})}  $$

(5) $$  {symm(C_{sxy}) = \bigwedge\limits_{i=0}^{c} symm(I_{sxyi})}  $$

(6) $$  { weight(C_{sxy}) = \min\limits_{i=0}^{c} weight(I_{sxyi})}  $$

#### Post-processing

An optional post-processing step is to automatically remove all inferred edges in the differential and/or consensus networks below a user-defined weight threshold. The exact value of this threshold should be chosen based on the input data and the edge weight normalisations of the original resources. For example, the differential weights could be indexed against the null distribution of values expected when the reference and condition-specific networks would represent equal replicates [6].

#### Fuzzy inference

The differential inference methods as described above can identify a rewiring event that is common to all conditions, as compared to one reference network. However, in some cases it might be beneficial to allow for one or more mismatches. Such a relaxed constraint enables for instance the retrieval of rewiring events that occur in three out of four conditions, thus allowing a more ‘fuzzy’ or less stringent mode of comparison.

For the calculation of consensus networks, similar relaxed criteria can be applied. In this case, it can be specified whether or not the reference network always needs to ‘match’ or not. If this is set to ‘true’, a consensus edge will always need support from the reference network specifically. Otherwise, all input networks are treated as equals.

### Implementation

Diffany is implemented in Java 1.6 and the code, released under an open-source license, contains extensive in-line documentation as well as detailed javadoc annotations^a^. JUnit tests ensure proper behaviour of the algorithms also after code refactoring. A GitHub repository provides version control, public issue tracking and a wiki with documentation. For instance, the framework could be extended by adapting more complex statistical scoring strategies [7, 12] into the ontology-based backbone. As this is a non-trivial task, we encourage others to contribute to this effort through the online GitHub repository.

The code base is structured in a modular fashion, with various methods for network cleaning, building and refining the ontology structure, applying custom edge filters, and so on. It is straightforward to extend the available functionality with additional network algorithms or filtering steps. By keeping semantics separate from functionality throughout the code, it becomes straightforward to create a custom ontology for any given project. On top of this core library, we have also implemented a Cytoscape plugin (‘app’) for the new Cytoscape 3 framework [20], providing an intuitive user interface and allowing straightforward integration with other network inference/analysis tools such as ClueGO [21], BINGO [22] or GeneMANIA [23]. Finally, a commandline interface supports large-scale bioinformatics studies through the generation of differential networks in straightforward tab-delimited file formats.

## Results

By design, the framework presented here can deal with any mixed input networks of negated edges, different edge weights, directed as well as symmetrical edges and a variety of edge types. Herein lays the main strength of our framework that is thus applicable to a wide range of comparative network studies.

### Genetic networks

**Fig. 4:**
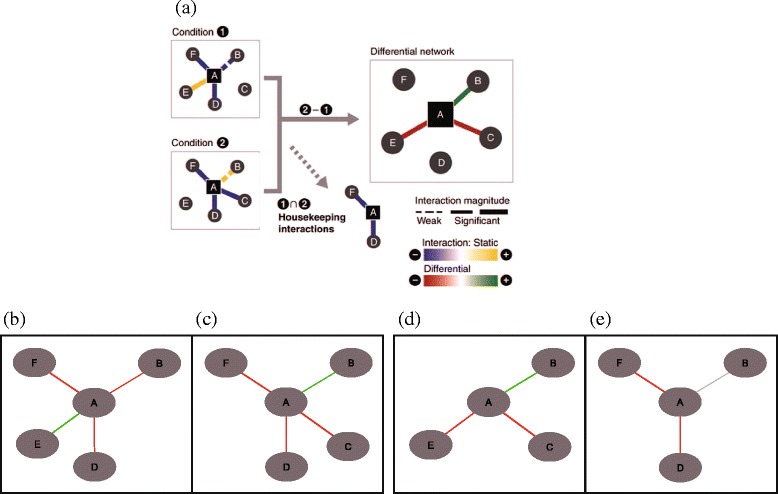
Artificial differential network of genetic interactions. A comparison of Diffany results with a previously published (artificial) differential network involving positive (alleviating) and negative (aggrevating) genetic interactions. **a**: The original picture by [6]. The reference network is denoted as ‘Condition 1’ and the condition-specific network as ‘Condition 2’. The differential network is displayed at the right, and the consensus network at the bottom (‘Housekeeping interactions’). **b**-**e**: The differential **d** and consensus networks **e** produced by Diffany from the same input data. Because they do not contribute to an enhanced understanding of the molecular rewiring, unconnected nodes are not included in the networks

To evaluate the implementation of our novel framework, we have applied it first to a small, artificial network available in previous literature (Fig. 4). Using the original inference as inspiration (Fig. 4a) to model the input networks (Fig. 4b-c), Diffany produced differential and consensus networks (Fig. 4d-e). Remarkably, compared to the inference of [6], the consensus network generated by Diffany contains one additional edge: the (weak) unspecified genetic interaction (gi) between A and B. Indeed, because our framework is ontology-driven, it can recognise the fact that ‘positive gi’ and ‘negative gi’ are both subclasses of the more general category ‘genetic interaction’. As a result, there is an edge of type ‘unspecified genetic interaction’ between nodes A and B in the consensus network.

In cases where such general, unspecified edges without polarity are unwanted, it is trivial to remove them from the network in a post-processing filtering step. However, we believe this additional information can be valuable when combined with the information in the differential networks themselves, as the presence or absence of such a generic consensus edge helps distinguishing between the three different cases as depicted in Fig. 1. Specifically, this generic regulatory edge provides evidence for the fact that both the reference and condition-specific network contain a regulatory edge between nodes A and B, but with opposite polarity, as is the case in the top example in Fig. 1. Given that the differential edge presents an increase in regulation, this means that the reference network contained a negative (down-) regulation, and the condition-specific network a positive (up-) regulation. When instead the consensus edge would not have this general, unspecified edge, as in the case of the bottom example in Fig. 1, this would mean that the condition-specific network simply did not have any link between the two nodes.

### Heterogeneous data

**Fig. 5:**
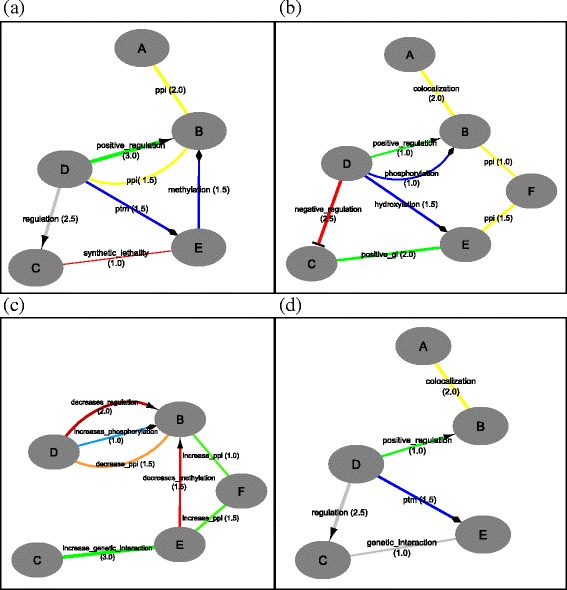
Artificial differential network of heterogeneous data. More complex calculation of differential (**c**) and consensus (**d**) networks from the reference (**a**) and condition-specific (**b**) networks. Notice how directionality, different edge types and weights can all be mixed freely in the networks

The second example presents the application of the Diffany inference tool to heterogeneous input networks, further illustrating the power of the Interaction Ontology. Here, a differential and a consensus network are generated from reference and condition-specific networks obtained through integrating various interaction and regulation types (Fig. 5). Notice how directionality, different edge types and weights can all be mixed freely in the networks.

### Mannitol-stress in plants

To demonstrate the practical utility of our framework, we have used Diffany to reanalyse a previously published experimental dataset measuring mannitol-induced stress responses in the model plant *Arabidopsis thaliana* [24]. In this study, nine-days-old seedlings were transferred to either control medium, or medium supplemented with 25 mM mannitol. At this developmental stage, the third true leaf is very small and its cells are actively proliferating. RNA from these young leaves was extracted at 1.5, 3, 12 and 24 h after transfer. The expression data were processed with robust multichip average (RMA) as implemented in BioConductor [25, 26]. Further, the Limma package [27] was applied to identify differentially expressed (DE) genes at two FDR-corrected P-values: 0.05 and 0.1, giving rise to two sets of DE genes for each time-point (Table 1 and Additional file 2).

**Table 1:** Number of differentially expressed genes per dataset

Time point	FDR 0.05	FDR 0.1
1.5 h	58	78
3 h	314	456
12 h	435	581
24 h	1500	1913
TOTAL	1638	2155

#### Input networks

To determine the set of genes (nodes) relevant to this study, we have first taken all differentially expressed genes across all time-points, using the strict 0.05 FDR threshold. Next, all the PPI neighbours of these genes were extracted from CORNET [28, 29] and added, with the exception of non-DE PPI hubs, as the inclusion of such hubs would extend our networks to irrelevant nodes. Analysis showed that for instance 10 % of all nodes account for 70 % of all PPI edges, and we have removed the bias towards such generic hubs by automatically excluding proteins with at least 10 PPI partners. Note that such hubs will still appear in the networks when they are differentially expressed themselves.

Subsequently, all regulatory neighbours of the extended node set were added, using both the AGRIS TF-target data [30] and the kinase-target relations from PhosPhAt [31]. From the kinase-target relations, hubs with at least 30 partners were excluded, removing mainly MAP kinase phosphatases (MKPs) which are involved in a large number of physiological processes during development and growth [32]. Finally, we also added DE genes from the second, less stringent result set (FDR cut-off 0.1), if they could be directly connected to at least one of the genes found up until that point. This approach allows us to explore also those genes that are only slightly above the strict 0.05 FDR cut-off, while reducing noise by excluding those that are not connected to our pathways of interest. In general, this two-step methodology as well as the hub filtering was found to produce more meaningful results. However, both steps are optional and can be removed from the pipeline when using the Diffany library in other studies.

The reference network was then defined by generating all PPI and regulatory edges between the node set as determined in the previous steps. All edges in the reference network were given weight one, a default value used when no overexpression is measured (yet). This resulted in a reference network of 1393 nodes and 2354 non-redundant edges, of which 56 % protein-protein interactions, 24 % TF regulatory interactions and 20 % kinase-target interactions.

Subsequently, each time-specific network was constructed by altering the edge weights according to the expression levels of the corresponding nodes/genes measured at that time point. All interactions with at least one significantly differentially expressed gene as interaction partner is thus down- or upweighted. To define differential expression, the less stringent criterium (0.1 FDR) is used here. For instance, the activation of a non-DE gene by a gene that is differentially expressed at that specific time point, would get a weight proportional to the fold change of that differentially expressed activator. By contrast, an edge would be removed (weight zero) when the edge does not fit the expression values at this time point, for instance when an activator is overexpressed but the target is underexpressed. This allows us to remove the interactions that, even though reported in the public interaction data, are probably not occurring in this specific context.

As a final result, the information on differentially expressed genes has now been encoded in the edge weights of the time-specific networks. By comparing them to the generic reference network, the Diffany algorithms will now be able to produce differential and consensus networks which depict the changes in expression values across the time measurements. In the following, we describe these results and provide interpretations that show-case how this type of analysis may lead to novel insights.

#### Differential network for one condition

With the statistically significant DE values translated into input networks, the differential networks can then be generated by either comparing the reference network to each time-specific network individually, or by comparing all time-specific networks against the reference network simultaneously.

**Fig. 6:**
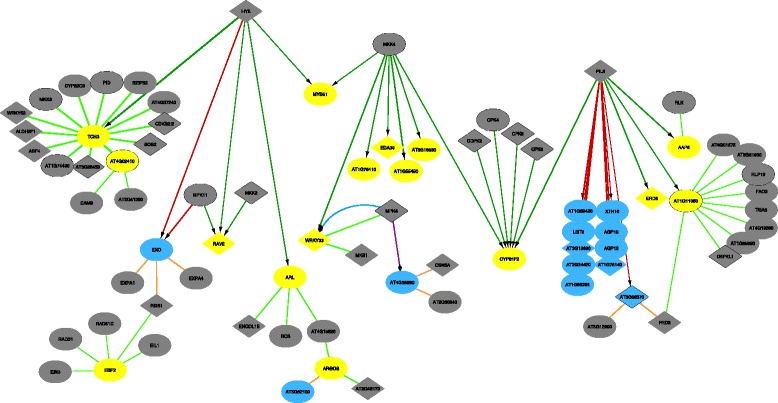
Mannitol-induced stress response at 1.5 h. Analysis of the mannitol-induced stress response, depicting the generated differential network at the 1.5 h time point: increase/decrease of regulation in dark green and red respectively, increase/decrease of PPI in light green and orange, increase/decrease in phosphorylation in blue and purple. It is important to note that in these differential networks the arrows point to rewiring events: a decrease of regulation for instance (red arrows) does not necessarily point to an inhibition, but may also indicate a discontinued activation. Diamond nodes represent proteins with a known phosphorylation site, and proteins with a kinase function are shown with a black border. Blue and yellow nodes identify underexpressed and overexpressed genes respectively

As an example of the first mode of comparison, Fig. 6 depicts the differential network after 1.5 hours, illustrating the rewiring events occurring in this short time frame after the induction of mannitol stress. At this early time point, it is rather unlikely that the expression of the DE genes was affected by subsequent transcriptional cascades. By including transcription factors upstream of the DE genes in the network even if they are not DE themselves, it is possible to identify new putative regulators as compared to previous analysis methods. For example, HY5 and PIL5 might be suitable candidates, as they contain a putative phosphorylation site and are thus likely to be posttranslationally regulated.

To further investigate the possibility that HY5 would be a transcriptional regulator under mannitol stress, we validated the Diffany results by measuring the expression level of the proposed HY5-target genes in the growing leaves of WT and HY5 loss-of-function mutants. These genes, except *ARL*, were all underexpressed in *hy5* mutants as compared to WT, confirming that HY5 is indeed involved in the regulation of the *MYB51*, *EXO*, *RAV2* and *TCH3* expression in growing Arabidopsis leaves (Additional files 3 and 4).

**Fig. 7:**
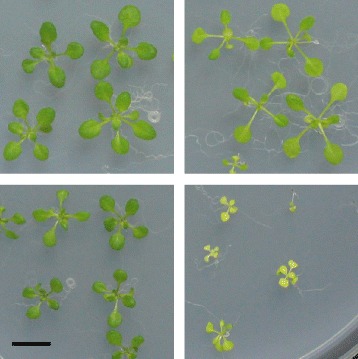
Phenotype of the hy5 mutant on mannitol-stress. Rosettes of WT (*left*) and hy5 mutants (*right*) on control medium (*top* panel) and mannitol-supplemented medium (*bottom* panel). Plants are 22 days old. Scalebar = 1 cm

To further explore if HY5 is involved in leaf growth regulation under mannitol stress, phenotypic analysis was performed on *hy5* mutants under both long term and short term mannitol treatment. The *hy5* seedlings were clearly hypersensitive to stress, with decreased leaf size under long term and short term stress, and showed complete bleaching upon long term mannitol stress (Fig. 7, Additional file 4). These biological results demonstrate that HY5, which has been identified with Diffany as a putative regulator of mannitol stress, might indeed be involved in the mannitol-responsive network in growing Arabidopsis leaves.

Next to the identification of new putative regulatory links, the differential PPI edges make it possible to understand complex formation under specific conditions. For example, the EBF2 sub-complex presents a nice example of how the induction of one protein is sufficient to increase the activity of a whole complex. The EBF2 is a stress-responsive E3-ligase involved in the posttranslational regulation of the ethylene-responsive factors EIN3 and EIL1 [33, 34]. In this differential network, EBF2 forms a complex with these two targets, which are induced by mannitol as well. However, some of the other members of the SCF-complex, such as CUL1, SKP1, ASK1 and ASK2, are missing from the differential network. As these SCF-complexes are involved in many cellular processes, their specificity being defined by the E3-ligase, we can speculate that the other members of the complex are highly abundant and not specific to mannitol-stress. Their automatic removal from the differential network thus allows the user to focus on the truly interesting genes for this specific stress condition.

#### Differential network for all conditions

**Fig. 8:**
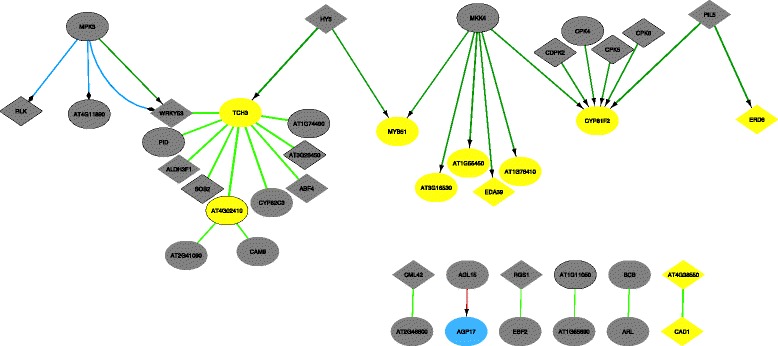
Mannitol-induced stress response across all time points (strict). Analysis of the mannitol-induced stress response, showing the differential network generated by comparing the reference network to all four time points simultaneously, and calculating the overall differential rewiring. Color coding as in Fig. 6

The second mode of comparison allows to simultaneously compare all condition-specific networks to one reference network. In this specific case, such an analysis models the stress-specific, but time-independent response. Fig. 8 shows these rewiring interactions. Strikingly, mainly the overexpressed genes (yellow nodes) remain differentially expressed throughout the time-course experiment, while this is only the case for a few of the underexpressed genes (blue nodes). This implies that in this context, the upregulation of genes is a more stable and long-term process.

For instance, the upregulation of *TCH3* by HY5 is present because *TCH3* is overexpressed at all time points and its upregulation by HY5 may thus play a significant role in the overall stress response. To validate this biologically, the expression level of *TCH3* and other previously mentioned HY5 target genes was measured in WT and *hy5* mutants, 24 h upon transfer to control or mannitol-supplemented medium (Additional file 4). While the induction of *TCH3*, *MYB51* and *ARL* could be clearly observed in WT plants, a more variable but less pronounced upregulation was observed in *hy5* mutants. Thus, HY5 might be involved in the regulation of *TCH3*, *MYB51* and *ARL* under mannitol, although it is probably not the sole regulator of these targets, but instead acts in parallel with other regulators previously identified in the early mannitol-response of growing Arabidopsis leaves [24, 35].

**Fig. 9:**
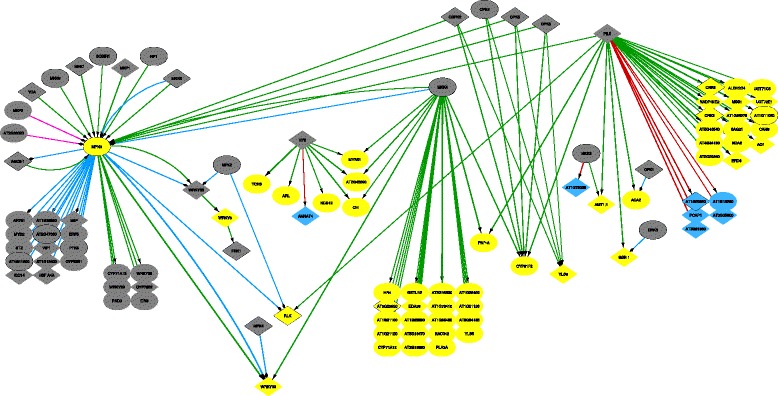
Mannitol-induced stress response across all time points, allowing for one mis-match per edge. Analysis of the mannitol-induced stress response, showing the differential network generated by comparing the reference network to all four time points but allowing a match when only three out of four time points share the same response. Color coding as in Fig. 6, pink arrows depict an increase in dephosphorylation. In this figure, only regulatory interactions are shown as the addition of PPI data would obscure the visualisation

Finally, we can apply a less stringent criterium to the inference of differential networks by only requiring that three out of four time points need to match for a rewiring event to be included in the differential network (Fig. 9). This results in more robust network inference, as the differential network would remain the same when some noise would be introduced at one of the time points. Additionally, this method provides a more complete view on the rewiring pathway occurring in response to osmotic stress in plants. All these different settings and options are also available when generating the differential networks through the Cytoscape plugin.

## Discussion and conclusion

We have developed an open-source framework, called Diffany, for the inference of differential networks from an arbitrary set of input networks. This input set always contains one reference network which represents the interactome of an untreated/unperturbed organism, while all other networks are condition-specific, each modelling the interactome of the same organism subjected to a specific environmental condition or stimulus. Differential networks allow focusing specifically on the rewiring of the network as a response to such stimuli, by modelling only the changed interactions. At the same time, interactions that remain (largely) the same are summarised in a ‘consensus’ network that provides insight into the basic interactions that are not influenced by changes of internal or external conditions. The analysis of these differential and consensus networks provides a unique opportunity to enhance our understanding of rewiring events occurring for instance when plants undergo environmental stress, or when a disease manifests in the human body. Further, the fact that the framework can compare an arbitrary number of condition-specific networks to one reference network at the same time, forms a powerful tool to analyse distinct but related conditions, such as different human diseases that may share a defected pathway, or various abiotic stresses influencing a plant in a similar fashion.

In comparison to previous work in the emerging field of differential network biology, Diffany is the first generic framework that provides data integration functionality in the context of differential networks. To this end, we have implemented an Interaction Ontology which enables seamless integration of different interaction types, provides semantic interpretation, and deals with heterogeneous input networks containing both directed and symmetrical relations. This ontology forms the backbone for the implementation of the network inference methods that produces differential networks. As in any Systems Biology study or application, a known challenge involves the issue of non-existing edges: an interaction may be missing from the network because it was experimentally determined that no association occurred, or it may simply be that there is a lack of evidence for the interaction, not actually excluding its existence. To deal with these cases, Diffany allows the definition of negated edges, which are explicit recordings of interactions that were determined not to happen under a specific condition.

To provide easy access to the basic functionality of inference and visualisation of differential and consensus networks, we have developed a commandline interface and a Cytoscape plugin. The Cytoscape plugin allows to generate custom differential networks as well as reproduce the use-cases described in this paper. All relevant code is released under an open-source license.

Finally, we have illustrated the practical utility of Diffany on a study involving osmotic stress responses in *Arabidopsis thaliana*. The resulting differential networks were found to be concise and coherent, modelling the response to mannitol-induced stress adequately. The analysis of these differential networks and a preliminary experimental validation has led to the identification of new candidate regulators for early mannitol-response, such as PIL5 and HY5, which likely contribute to the fast transcriptional induction of mannitol-responsive genes. Further detailed biological validation, including for instance ChIP experiments and experimental systems biology approaches, are necessary to confirm the role of HY5 in this context and fully unravel the early stress-induced rewiring events of this complex regulatory network.

## Endnote

^a^ API at http://bioinformatics.psb.ugent.be/supplementary_data/solan/diffany/.

## Additional files

Additional file 1
**Overview of the Diffany framework.** Overview of the Diffany framework and its typical usage in a specific experiment involving the perturbation of an interactome under one or more conditions. (DOCX 183 KB)

Additional file 2
**List of differentially expressed genes.** Dataset of differentially expressed genes, as originally published by [24]. Here, those genes are listed that are differentially expressed in at least one of the 4 time points and in either the more (FDR < 0.05) or less (FDR < 0.1) stringent dataset. This file also depicts the overlap of genes at the different time points. (XLSX 514 KB)

Additional file 3
**Experimental methodology.** Methodological details of the experiments performed on the putative HY5 regulator. (DOCX 22 KB)

Additional file 4
**Figure showing the experimental validation of the putative HY5 regulator.** Detailed analysis of hy5 mutants and WT lines when exposed to mannitol-induced stress, comparing both leaf area as well as expression levels of putative HY5-target genes such as *TCH3* and *MYB51*. (DOCX 472 KB)
