# Supplementary material for: Diffany: an ontology-driven framework to infer, visualise and analyse differential molecular networks
Source: BMC Bioinformatics. 2016 Jan 5;17:18. doi: 10.1186/s12859-015-0863-y (PMC4700732; doi:10.1186/s12859-015-0863-y)
Supplement: Additional file 1 — Overview of the Diffany framework. Overview of the Diffany framework and its typical usage in a specific experiment involving the perturbation of an interactome under one or more conditions. (DOCX 183 KB) [file 12859_2015_863_MOESM1_ESM.docx]

**Overview of the Diffany framework and its typical usage** in a specific experiment involving the perturbation of an interactome under one or more conditions.


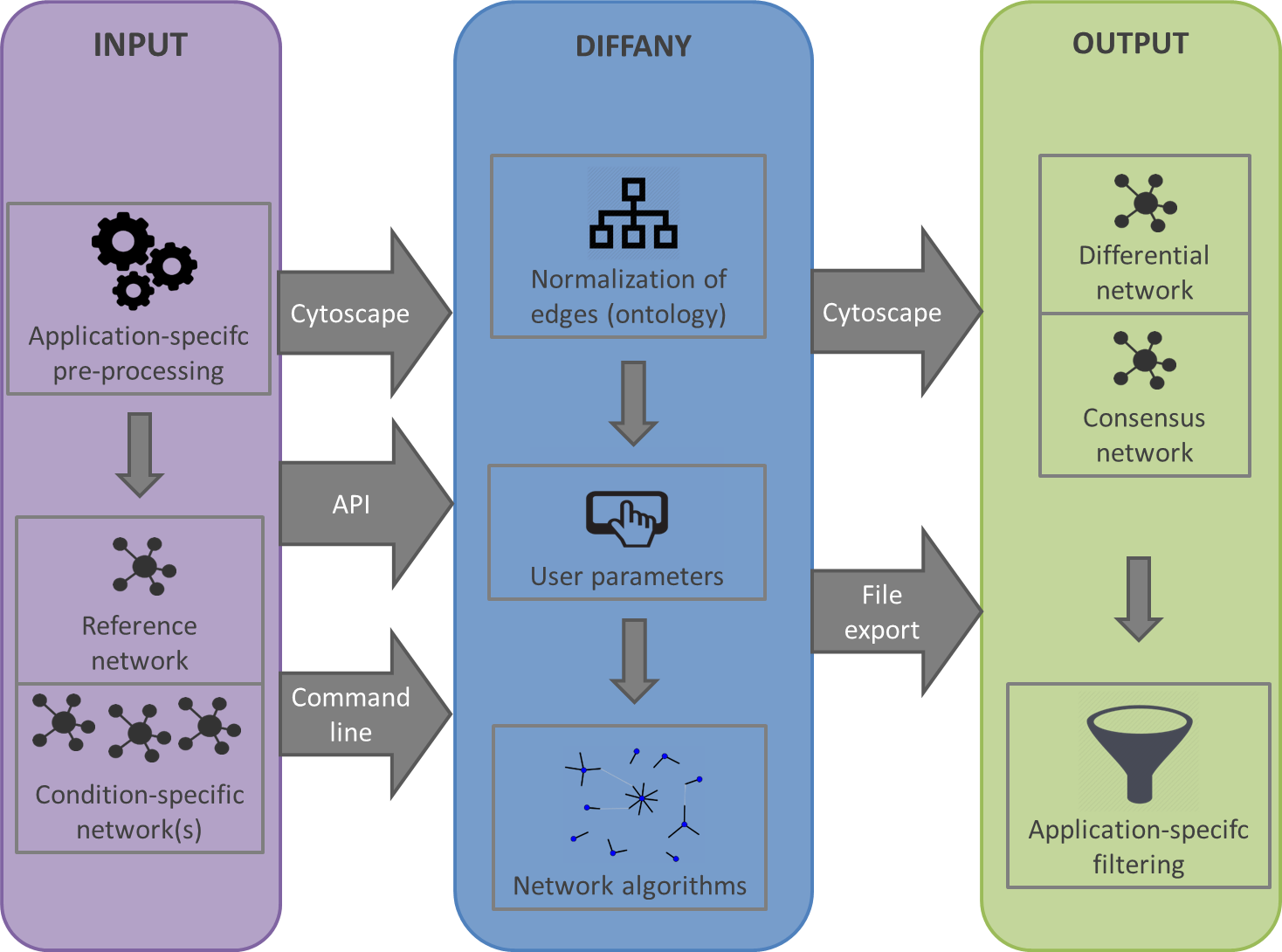


**Input**

- Pre-processing to define networks that are relevant to the specific application
- Reference network: unperturbed interactome
  - Can have a heterogeneous set of interactions (edges)
  - Edges can have weights and are directed or symmetrical
- One or more condition-specific networks: perturbed interactome
  - Similar properties as for the reference network

**Diffany framework**

- Normalization of edges using the interaction ontology
  - Default ontology available in the framework
  - Custom ontology can be encoded
- User parameters: depend on application/user requirements
  - 1-1 or 1-all comparison
  - (in case of 1-all comparison) : fuzzy inference or not
- Network algorithms
  - For each node pair and for each root category of the interaction ontology,
    calculate the relevant differential and consensus edges

**Output**

- Differential network: rewiring events caused by the perturbation
- Consensus network: interactions not influenced by the perturbation
- Filtering according to the application/user requirements
  - remove all inferred edges below a user-defined weight threshold
  - remove generic edges
  - …
