# Supplementary material for: Diffany: an ontology-driven framework to infer, visualise and analyse differential molecular networks
Source: BMC Bioinformatics. 2016 Jan 5;17:18. doi: 10.1186/s12859-015-0863-y (PMC4700732; doi:10.1186/s12859-015-0863-y)
Supplement: Additional file 3 — Experimental methodology. Methodological details of the experiments performed on the putative HY5 regulator. (DOCX 22 KB) [file 12859_2015_863_MOESM3_ESM.docx]

**Plant lines**

The hy5 mutant was obtained from the SALK collection and is in Col-0 background.

***In vitro* plant growth conditions**

Seedlings were grown *in vitro* on half-strength Murashige and Skoog medium ([Murashige and Skoog, 1962](#_ENREF_44)) containing 1% sucrose at 21˚C under a 16-h day (110 μmol m^-2^ s^‑1^) and 8-h night regime. For long term experiments where no transfer was needed, 9 g/L agar was added to the medium. For short term experiments involving transfer, 6,5 g/L agar was used and the growth medium was overlaid with nylon mesh (Prosep, Zaventem, Belgium) of 20 µm pore size to facilitate transfer. For expression analysis and growth experiments, 10 seeds were equally distributed on a 14cm diameter petri dish. The *hy5* mutant was always grown together with the appropriate control on 1 plate to enable correct comparisons.

At 15 days after stratification, when the third leaf is expanding, the mesh with seedlings was transferred to plates with 1/2MS medium or with 1/2MS containing 25 mM D-mannitol (plant culture tested, Sigma).

**Growth analysis**

Growth analysis was performed on the third true leaf harvested 5 days upon transfer. For each experiment, about 10 leaves were photographed and pictures were used to measure leaf area using ImageJ v1.37o (NIH; http://rsb.info.nih.gov/ij/).

**Expression analysis**

24h after transfer, the third leaves of 10 seedlings were frozen in liquid nitrogen, and ground with a Retsch machine and 3-mm metal balls. RNA was extracted with TriZol (Invitrogen) and further purified with the Rneasy Mini Kit (Qiagen). DNA digestion was done on-column with Rnase-free DNase I (Promega). For cDNA synthesis, the iScript cDNA Synthesis Kit (Biorad) was used according to the manufacturer’s instructions using 1 μg of RNA. qRT-PCR was done on a LightCycler 480 (Roche Diagnostics) in 384-well plates with LightCycler 480 SYBR Green I Master (Roche) according to the manufacturer's instructions. Melting curves were analyzed to check primer specificity. Normalization was done against the average of housekeeping genes AT1G13320
AT2G32170, AT2G28390; ΔCt = Ct (gene) – Ct (mean (housekeeping genes)) and ΔΔCt = ΔCt(control line)- ΔCt(line of interest). Ct refers to the number of cycles at which SYBR Green fluorescence reaches an arbitrary value during the exponential phase of amplification. Primers were designed with the QuantPrime website ([Arvidsson et al., 2008](#_ENREF_6)). Primers used in this study are: *MYB51*: GCCCTTCACGGCAACAAATG and GGTTATGCCCTTGTGTGTAACTGG, *RAV2*: TGGGAAGCTAAACCGTCTCGTG and ACGGTGACGGTAACGGAAAGTG, *TCH3*: TCGACAAGAATGGTGATGGTTCC and TCACCGATTGAACGCATCATGG, *ARL*: TCAGCTCCAGCTCCGATTATGG and TGGACTGTTCTGCGACGACAATG, *EXO*: ACCAAACAATGACGTGGGACTCG and AAAGGGTTCGTTGCGGTTCCAG
