# Supplementary material for: Diffany: an ontology-driven framework to infer, visualise and analyse differential molecular networks
Source: BMC Bioinformatics. 2016 Jan 5;17:18. doi: 10.1186/s12859-015-0863-y (PMC4700732; doi:10.1186/s12859-015-0863-y)
Supplement: Additional file 4 — Figure showing the experimental validation of the putative HY5 regulator. Detailed analysis of hy5 mutants and WT lines when exposed to mannitol-induced stress, comparing both leaf area as well as expression levels of putative HY5-target genes such as TCH3 and MYB51. (DOCX 472 KB) [file 12859_2015_863_MOESM4_ESM.docx]

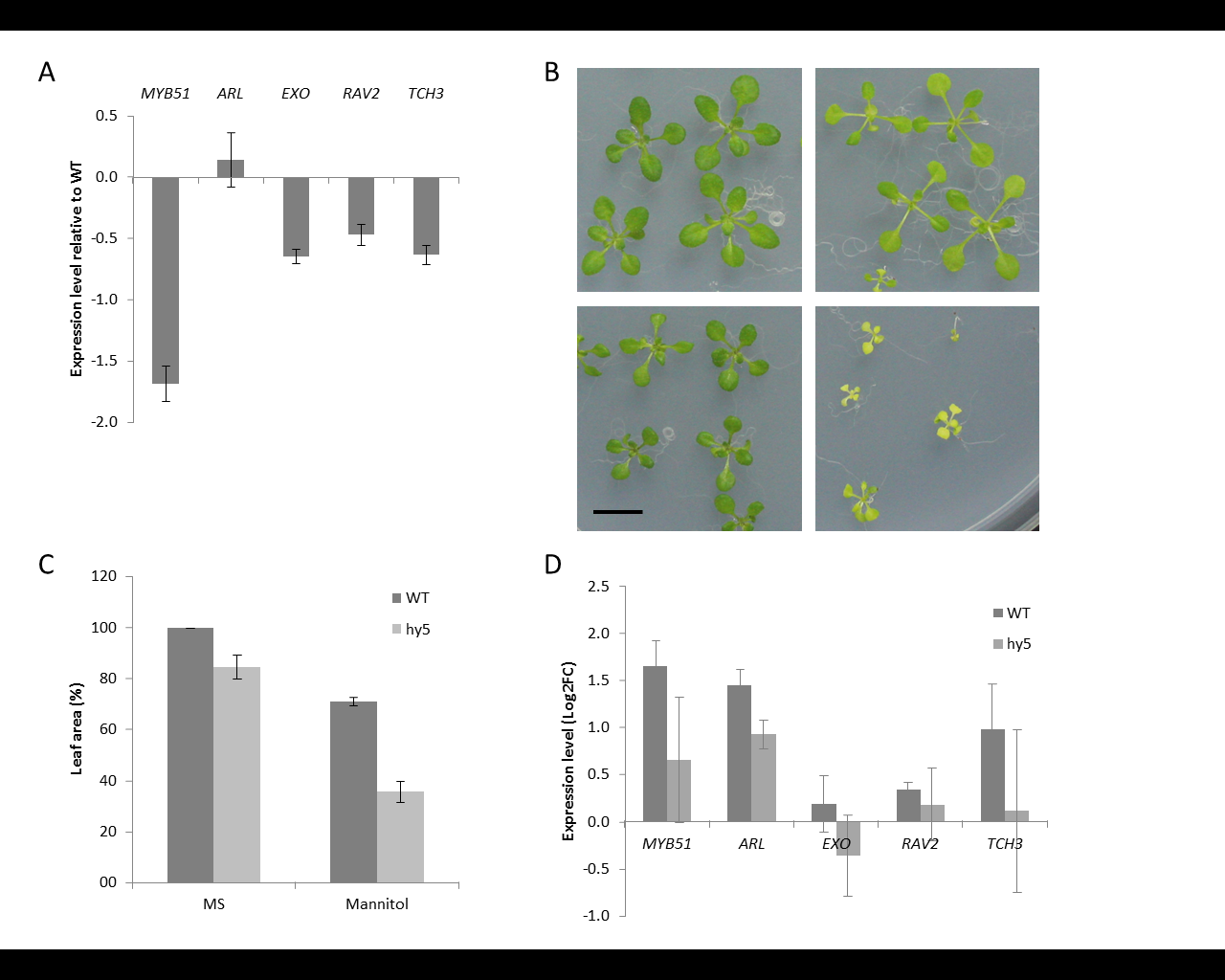


(A) Expression of the putative HY5-target genes in the hy5 mutant relative to WT. Expression was measured in expanding 3rd leaves and values are normalized to their expression in the WT line in the same condition. Error bars indicate standard errors of 3 repeats with 10 leaves per repeat. (B) Rosettes of WT (left) and hy5 mutants (right) on control medium (top panel) and mannitol-supplemented medium (bottom panel). Plants are 22 days old. Scalebar = 1 cm. (C) Leaf area measurements (3rd leaf) at day 20 of hy5 mutants and WT upon transfer at 15 days after stratification to control or mannitol-containing medium. While mannitol decreases the leaf size in WT by 29%, this mannitol-induced reduction reaches on average 49% in hy5 mutants. Error bars indicate standard errors. Three biological repeats were performed with 10 leaves per repeat. (D) Induction of the putative HY5-target genes upon transfer to mannitol-supplemented medium in WT and hy5 plants. Expression was measured in expanding 3rd leaves and values are normalized to their expression level under control conditions (=0). Error bars indicate standard errors of 3 repeats with 10 leaves per repeat.
